# Effect of Nicotinamide in Skin Cancer and Actinic Keratoses Chemoprophylaxis, and Adverse Effects Related to Nicotinamide: A Systematic Review and Meta-Analysis

**DOI:** 10.1177/12034754221078201

**Published:** 2022-02-08

**Authors:** Laurence Mainville, Anne-Sophie Smilga, Paul R. Fortin

**Affiliations:** 1 4440 Faculty of Medicine, Laval University, Quebec, Canada; 2 177453 Infectious and Immune Diseases, Centre de recherche du CHU de Québec – Université Laval, Quebec, Canada; 3 Division of Rheumatology, Department of Medicine, CHU de Québec – Université Laval, Quebec, Canada

**Keywords:** nicotinamide, niacinamide, chemoprophylaxis, chemoprevention, skin cancer, actinic keratosis, adverse effect, oncology, basal cell carcinoma, cutaneous squamous cell carcinoma, melanoma

## Abstract

**Background:**

Oral nicotinamide is recommended in individuals with a field of cancerization or with ≥1 previous cutaneous squamous cell carcinoma (cSCC).

**Objective:**

To evaluate the effect of nicotinamide in prevention of skin cancers.

**Methods:**

We conducted a systematic review and meta-analysis of randomized controlled trials to evaluate the effect of nicotinamide. We used Medline, EMBASE, CENTRAL, and Web of Science databases from their inception to October 2020 to search the following concepts: “nicotinamide”; “randomized controlled trial” (validated filters). Two independent reviewers screened titles and abstracts for intervention and study design before searching full texts for eligibility criteria. To be eligible, ≥1 outcome had to be covered. We used a standardized collection grid to complete data extraction in duplicate. The primary outcome was skin cancers (all types). Secondary outcomes were basal cell carcinomas (BCCs); cSCCs; actinic keratoses; melanomas; digestive, cutaneous, and biochemical adverse effects (AEs). Subgroup analyses were planned *a priori*.

**Results:**

We screened 4730 citations and found 29 trials (3039 patients) meeting inclusion criteria. Nicotinamide was associated with a significant reduction in skin cancers compared to control (rate ratio 0.50 (95% CI, 0.29-0.85; *I* ^2^ = 64%; 552 patients; 5 trials); moderate strength of the evidence). Heterogeneity was explained by risk of bias. Nicotinamide was associated with a significant reduction in BCCs and cSCCs, and increased risk of digestive AEs.

**Conclusion:**

Oral nicotinamide should be considered in healthy patients or organ transplant recipients with history of skin cancer (GRADE: weak recommendation; moderate-quality evidence), in particular of BCC and cSCC.

## Introduction

Exposure to UV light and immunosuppression are known risk factors for skin cancers.^1^ Among Canadians, basal cell carcinoma (BCC) and cutaneous squamous cell carcinoma (cSCC) are the most frequently diagnosed cancers.^2^

Nicotinamide is a form of vitamin B3. It is thought that its role in chemoprophylaxis is through reparation of DNA damage and reduction of immunosuppression due to UV exposure.^3-7^ Recent recommendations from 2018 and 2020 published in the Journal of the American Academy of Dermatology support the use of oral nicotinamide 500 mg twice daily in patients with a field of cancerization (diffuse actinic keratoses/in situ cSCCs) or >1 previous cSCCs.^8,9^ This recommendation is based on the results of one RCT conducted in 386 immunocompetent Australians.^10^ In a systematic review evaluating the effect of chemopreventive interventions in solid organ transplant recipients, nicotinamide was not shown to be different from placebo.^11^ High dose nicotinamide (>3 g/day) can cause reversible hepatotoxicity; it was otherwise shown to be safe and well tolerated.^8^ The role of nicotinamide in chemoprophylaxis of melanocytic tumors is biologically plausible but remains to be clarified in clinical trials.^12^ Knowledge on pharmacokinetics of topical nicotinamide is developing in translational research. Nicotinamide and its lipophilic analog methyl nicotinate were similarly absorbed in ex vivo human skin, and in vivo dermal delivery of nicotinamide was greater with a binary vehicle of propylene glycol and linolenic acid.^13-15^

This systematic review and meta-analysis aimed to assess the effect of nicotinamide for skin cancer chemoprophylaxis in a large population of patients regardless of immunosuppression status.

## Study Objectives

The primary objective was to assess the effect of nicotinamide in comparison with placebo, vehicle, standard of care, no treatment or any other treatment with neutral or weak effect in skin cancer chemoprophylaxis. Secondary objectives were to evaluate the effect of nicotinamide in chemoprophylaxis of (1) BCCs, (2) cSCCs, (3) AK, and (4) melanoma, and the occurrence of (5) digestive, (6) cutaneous, and (7) biochemical AEs due to nicotinamide.

## Methods

### Study Design

The protocol was written according to PRISMA-P recommendations.^16^ It was submitted in PROSPERO (CRD42021223823). The methods follow the *Cochrane Handbook for Systematic Reviewers* (version 6.1, 2020).^17^ Results were reported according to the PRISMA statement.^18^

### Search Strategy

We conducted the search strategy using Medline (PubMed), EMBASE (Embase), CENTRAL, and Web of Science databases from their inception to October 2020. The search strategy was validated with an information specialist. We did not limit our search to individuals with a history of skin cancers in order to include all trials using nicotinamide and report incidental data on skin cancers. The strategy used for Medline (Pubmed) is presented in eTable1 in the Supplemental material. Filters validated to research RCTs were used.^19^ References of included studies and previous reviews on the subject were checked for studies that meet our eligibility criteria. Companion articles of eligible studies were considered for inclusion.

### Eligibility Criteria

Individual studies considered eligible were published and peer reviewed RCTs. They assessed the effect of nicotinamide compared to placebo, vehicle, standard of care, no treatment or any other treatment with neutral or weak effect in prevention of skin cancers. We aimed to include citations in primary, secondary, and tertiary prevention, namely studies conducted in individuals without previous skin cancers or AK, and with previous or ongoing skin cancers or AK.^20^ The dose of nicotinamide had to be specified. Trials with co-interventions were considered eligible. All routes of administration were considered eligible to keep broad inclusion criteria. Quantitative data on AEs had to be reported. At least one outcome had to be covered. No restriction was applied for language, year of publication, and risk factors for skin cancer.

### Outcome Measures

The primary outcome was the number of new skin cancers, all types of skin cancers combined. Secondary outcomes were the number of new BCCs, the number of new cSCCs, the mean number of AK, the occurrence of melanoma, the occurrence of digestive, cutaneous, and biochemical AEs. All outcomes were evaluated at date of last follow-up. We used the authors’ definitions for AK, and skin cancers confirmed with histology. For biochemical AEs, we considered all laboratory tests and used normal values as defined by the authors. For digestive and cutaneous AEs, all reported signs and symptoms were taken into account. In presence of repeated measures, we considered the most distant measure that included the intervention period for analysis.

### Study Selection

Two independent reviewers (L.M. and A.-S.S.) screened titles and abstracts for intervention and study design. The full-text of selected citations was searched in duplicate (L.M. and A.-S.S.) for eligibility criteria. These steps were realized using *Covidence Systematic Review Software* (*Veritas Health Innovation*, Melbourne, Australia). All disagreements were resolved by consensus between L.M. and A.-S.S. We used an online translator to screen studies published in non-French or English language.

### Data Extraction

Data extraction was conducted using a standardized collection grid retrieving studies and participants characteristics; interventions received; number of skin cancers; follow-up per participant; mean number of AK and standard deviation; number of patients with melanoma; digestive, cutaneous and biochemical AEs; data on risks of bias and study sponsorship. Data were collected at the date of last follow-up. The AE with the highest number of events was retained in order to avoid multiple reports of a single participant within a dichotomous variable. For information, we specified the AEs retained per citation (eTable 2 in the Supplemental material). For crossover RCTs, we extracted the latest available data after first treatment and wash-out periods. For graphic data, we extracted relevant information by hand-measurements. We planned to contact authors up to two times in case of missing information regarding primary outcome but did not need to. Data extraction was completed in duplicate (L.M. and A.-S.S.).

### Data Synthesis and Statistical Analyses

Pooled counts of rare events are based on rates, namely on counts per amount of time during which each participant was followed. They are presented in rate ratios transformed to allow for statistical analyses (SE of ln[rate ratio]), with a 95% CI.^21^ Pooled continuous data are presented as mean differences, and pooled dichotomous data are presented as relative risks, with a 95% CI. A meta-analysis of the results was conducted for all outcomes using Review Manager, version 5.4.1 (RevMan, Copenhagen: The Nordic Cochrane Center, The Cochrane Collaboration, 2020). Meta-analysis was based on random-effects models, and the inverse variance method (continuous and dichotomous variables) or the generic inverse variance (counts of rare events).^22^ If the numerator equalled zero, a value of 0.5 was added to allow statistical analysis.

Statistical heterogeneity between studies was assessed using *I* ^2^ statistic.^23^ *A priori* planned subgroup analyzes were used to explain known or potential sources of heterogeneity based on: (1) the route of administration of nicotinamide [topical or enteral or intravenous or other]; (2) the daily dose of nicotinamide [<1 g or 1 g and more]; (3) the duration of the intervention [<1 year or 1 year and more]; (4) the risk of skin cancer [general risk or predisposing condition]; (5) co-interventions [absence or presence]; (6) the type of comparator [active or not active]; (7) risk of bias [low, high or some concerns]. We interpreted the heterogeneity between study data with the global, subgroup and *I* ^2^ for subgroup differences statistics.

### Internal and External Validity Assessment

Risk of bias was assessed in duplicate (L.M. and A.-S.S.) using the 5 domains of the Cochrane Risk of Bias tool (Rob2).^24^ An outcome was at high risk of bias if ≥1 domain was at high risk or if we had some concerns regarding multiple domains. We had some concerns regarding the risk of bias if ≥1 domain was rated with some concerns. Publication bias was assessed with funnel plots, when ≥ 10 trials were reported. Sources of funding were identified. The quality of the evidence was assessed in duplicate (L.M. and A.-S.S.). It was considered high, moderate, low or very low using the *Grades of Recommendation, Assessment, Development, and Evaluation* (GRADE).^25^

## Results

**Figure 1:**
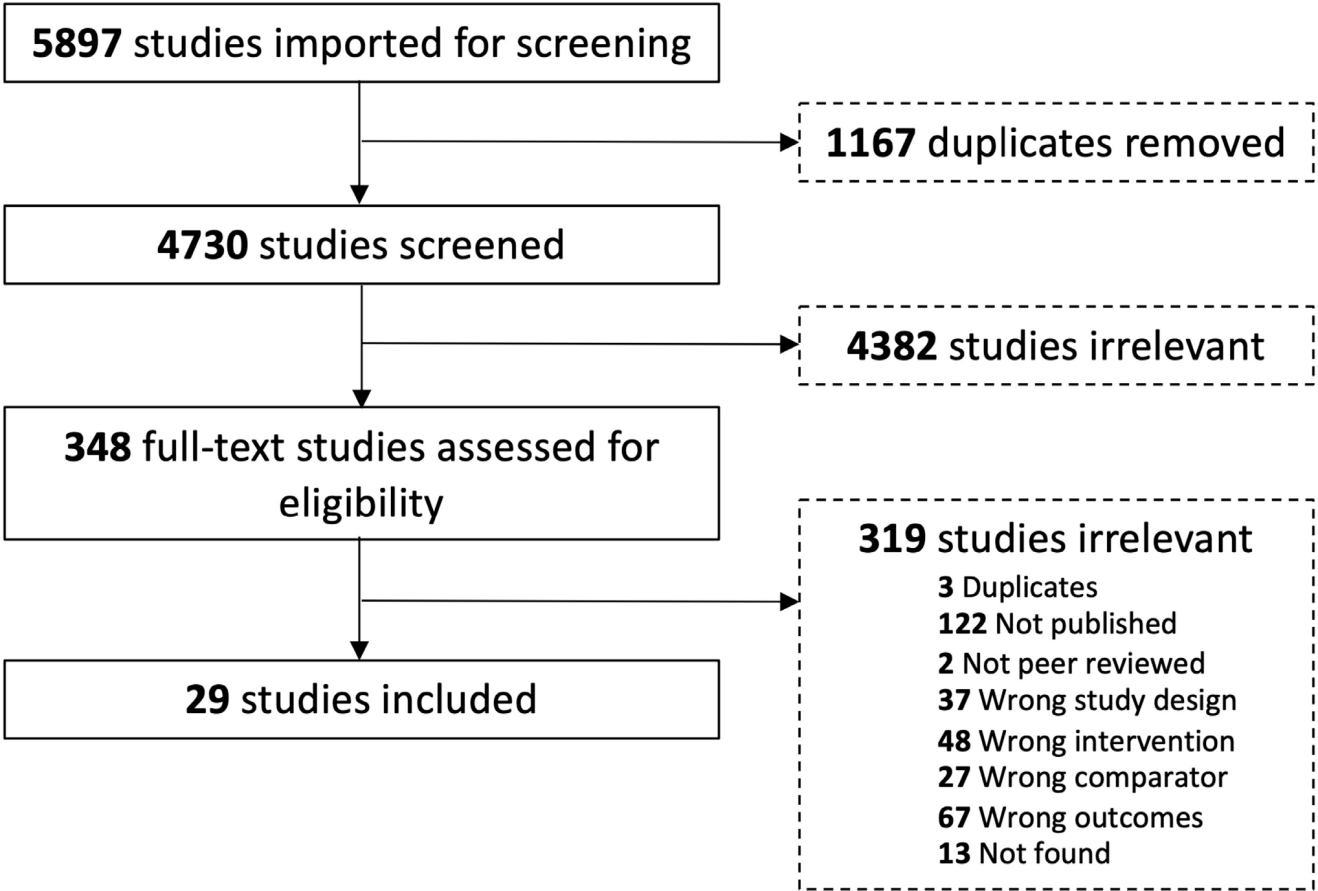
Flow diagram of trials.

Of the 5897 studies identified from electronic and hand-searches, we included 29 RCTs that enrolled 3039 participants (range 17 to 552) (Table 1; Figure 1). Publication year ranged from 1979 to 2020. The mean age of enrolled patients ranged from 10 to 75 years. Eleven of the 29 included trials were multicentered.^10,26-35^ All included trials were published in English. Five trials were conducted in Australia,^10,35-38^ including 4 by the same research group. All trials were in parallel groups, except for one with a crossover design.^35^ All trials with data on skin cancers were independently financed,^10,36,38,39^ except one that did not mention funding source.^37^

**Table 1:** Characteristics of Included Studies.

Trial	NAM: Control	Indication of studied intervention^a^	Nicotinamide posology	Co-intervention	Treatment duration
Chouinard 1979	8 + 9:8	Depression	1.5 g po die	Tryptophan +/-Imipramine	1 month
Hulshof 1987	24:24	Tinnitus	70 mg po die	None	1 month
Fivenson 1994	12:6	Bullous pemphigoid	500 mg po tid	Oral tetracycline	1 year
Shalita 1995	38:38	Acne	4% gel bid	None	2 months
Jonas 1996	31:29	Osteoarthritis	500 mg po 6 x/d	None	3 months
Gale 2004	276:276	Type 1 diabetes	1.2 g/m^2^ po die	None	5 years
Sun 2007	45:44	Alzheimer	10 mg po die	Multivitamin	6 months
Young 2009	8:9	Hyperphosphatemia in peritoneal dialysis	750 mg po bid	None	2 months
Jerajani 2010	124:122	Normal skin	4% lotion	None	2 months
Moloney 2010	13:17	Tertiary prevention of AK in healthy adults with ≥4 AK	1% gel bid	None	6 months
Shahbazian 2011	24:24	Hyperphosphatemia in hemodialysis	1 g po die	None	2 months
Allam 2012	30:30	Hyperphosphatemia in hemodialysis	1 g po die	Calcium	2 months
Surjana 2012	39:37	Tertiary prevention of AK in healthy adults with ≥4 AK	500 mg po die/bid	None	4 months
Khodaeiani 2013	40 :40	Acne	4% gel bid	None	2 years
Pop-Busui 2013	22:22	Diabetes	750 mg po bid	Allopurinol, αlipoic acid	2 years
Fabbrocini 2014	24:24	Seborrheic dermatitis	4% cream die	None	3 months
Chen 2015	193:193	Tertiary prevention of BCC/SCC/AK in healthy adults with ≥2 NMSC in previous 5 years	500 mg po bid	None	6 months
Watanabe 2015	52 :52	Androgenetic alopecia	0.1% lotion bid	None	6 months
Chen 2016	11:11	Tertiary prevention of BCC/cSCC/AK in immunocompromised kidney transplant recipients with ≥2 NMSC in previous 12 months	500 mg po bid	None	1 year
El Borolossy 2016	30:30	Hyperphosphatemia in children on hemodialysis	100 mg po die or bid	Calcium	6 months
Kasliwal 2016	96:95	Dyslipidemia	7 mg po bid	Powders of red yeast rice, grape seed extract, black pepper, B9	3 months
Drago 2017	19:19	Tertiary prevention of AK in immunocompromised liver/kidney transplant recipients with ≥1 untreated AK	250 mg po tid	None	6 months
Lenglet 2017	49:51	Hyperphosphatemia in hemodialysis	0.5-2g po die	None	6 months
Rucklidge 2018	47:46	Attention deficit hyperactivity disorder	72 mg po die	Micronutrients	2 months
Caetano 2019	44 + 44:44	Oily skin	4% cream	Cleanser +/-topical salicylic acid	2 months
Ix 2019	104:101	Hyperphosphatemia in chronic kidney disease	750 mg po bid	Placebo +/-Lanthanum carbonate	1 year
El Ters 2020	18:18	Autosomal dominant polycystic kidney disease	30 mg/kg/d po	None	1 year
Hui 2020	30:27	Glaucoma	1.5 g po bid	None	3 months
Liu 2020	49:49	Hyperphosphatemia in hemodialysis	0.5-1.5g po die	None	1 year

Abbreviations: AK, actinic keratoses; BCC, basal cell carcinoma; cSCC, cutaneous squamous cell carcinoma;d, day; g, gram; mg, milligram; NMSC, nonmelanoma skin cancer;po, per os.

^a^We aimed to evaluate the effect of nicotinamide in primary (measures to reduce behaviors related to an increase in risk of skin cancer), secondary (measures to detect and treat diseases at an early stage), and tertiary prevention of skin cancers (measures to prevent recurrences after skin cancer is diagnosed); thus, all indications of nicotinamide were considered.

The study population in 5/29 included trials consisted in patients with previous BCCs and cSCCs or untreated AK.^10,36-39^ Indication for nicotinamide therapy varied depending on immunosuppression status: from ≥2 keratinocyte carcinomas in previous 12 months (transplant recipients)^36^ to 5 years (healthy patients)^10^; and from ≥1 (healthy patients)^39^ to 4 untreated AK (transplant recipients).^37,38^ Other indications for study therapy were heterogeneous, including hyperphosphatemia and acne, as our population was not limited to individuals with a history of previous skin cancer and 24/29 trials were included based on one of the outcomes on adverse effects (Table 1). Nicotinamide was administered with ≥1 co-intervention in 10/29 trials (all neutral on skin cancer).^26,27,31,32,34,40-44^ In 10/29 trials, the comparators had neutral effect on skin cancer.^26-28,31,33,34,42,43,45,46^

## Primary Outcome

**Figure 2:**
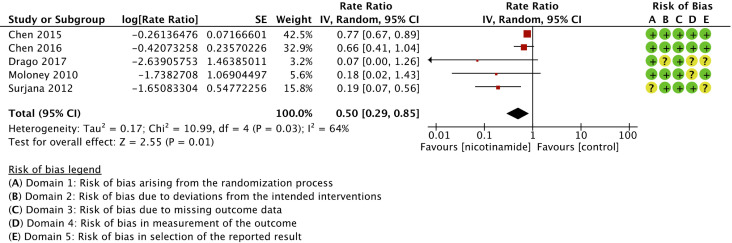
Forest plot and risk of bias for the effect of nicotinamide versus control in skin cancer chemoprophylaxis.

**Figure 3:**
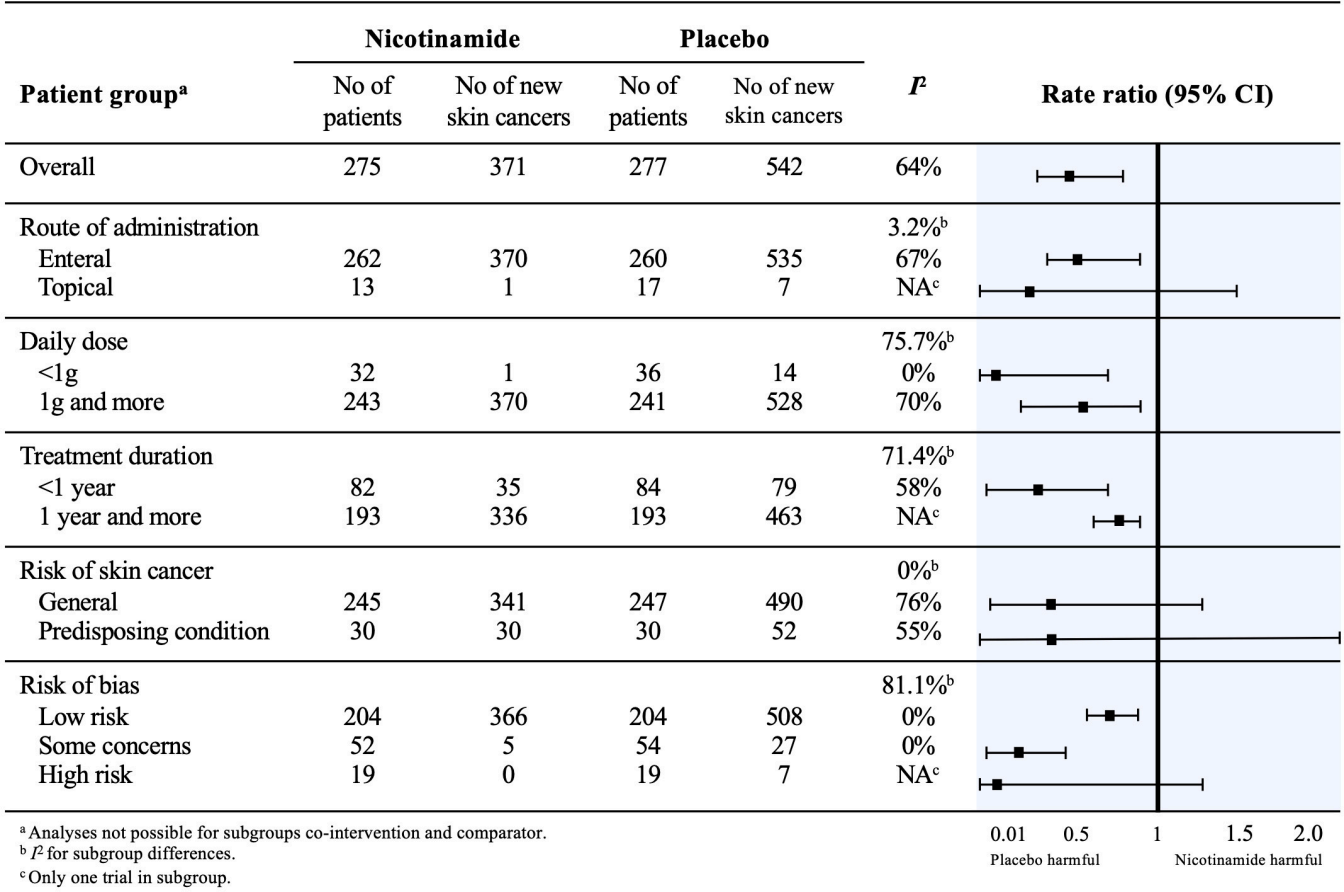
Subgroup analyses for the effect of nicotinamide versus control in skin cancer chemoprophylaxis.

Nicotinamide was associated with a significant reduction in skin cancers compared to control (rate ratio 0.50 (95% CI, 0.29-0.85; *I* ^2^ = 64%; 552 patients; 5 trials))^10,36-39^ (Figure 2). Risk of bias for skin cancers is presented in Figure 3. Regarding melanoma, only 1/5 trials reported sufficient person-time data to be included in the primary outcome analysis, namely number of individuals per groups and time of follow-up.^38^ Three of five trials reporting BCCs and cSCCs were not designed to evaluate skin cancers.^37-39^ Consequently, unplanned skin cancer reports and analyses increased the risk of bias in outcome measurements (RoB2, domain 4) and selective reporting (RoB2, domain 5), although not sufficiently to affect quality of the evidence. We graded the overall strength of the evidence as moderate (Table 2), due to indirectness of measures taken from trials studying nicotinamide in tertiary prevention.

**Table 2:** Summary of Findings.

	Quality assessment					Summary of findings		
No of studies (participants)	Risk of bias	Consistency	Directness	Precision	Publication bias	Relative effect (95% CI)^a^	Absolute effect (95% CI)^b^	Quality, GRADE
Skin cancers: 5 (552)	No serious limitation^c^	No inconsistency^d^	Serious indirectness (−1)^e^	No serious imprecision	Not detected^f^	Rate ratio = .50 (0.29, 0.85)	−1.22 per person-year (-1.83, −0.62)	⊕⊕⊕⊝, moderate
									BCC: 5 (552)	No serious limitation^c^	Serious inconsistency (−1)^g^	Serious indirectness (−1)^h^	No serious imprecision	Not detected^f^	Rate ratio = .46 (0.22, 0.95)	−0.74 per person-year (-1.13, −0.35)	⊕⊕⊝⊝, low
cSCC: 5 (552)	No serious limitation^c^	No inconsistency^i^	Serious indirectness (−1)^e^	No serious imprecision	Not detected^f^	Rate ratio = .48 (0.26, 0.88)	−0.53 per person-year (-1.03, −0.04)	⊕⊕⊕⊝, moderate
AK: 3 (492)	No serious limitation	Serious inconsistency (−1)^j^	Serious indirectness (−1)^h^	Serious imprecision (−1)^i^	Not detected^f^	—	−4.48 (-12.68, 3.73)	⊕⊝⊝⊝, very low
Melanoma: 2 (416)	No serious limitation^c^	No inconsistency	Serious indirectness (−1)^e^	Serious imprecision (−1)^l^	Not detected^f^	RR = .89 (0.29, 2.79)	0.43% fewer melanoma (3.51 fewer to 2.65 more)	⊕⊕⊝⊝, low
GI AE: 21 (1859)	Serious limitations (−1)^m^	No inconsistency	Serious indirectness (−1)^o^	Serious imprecision (−1)^l^	Unlikely	RR = 1.78 (1.30, 2.45)	5.5% more GI AEs (3.1%, 8.0% more)	⊕⊝⊝⊝, very low
Cutaneous AE: 19 (1805)	Serious limitations (−1)^n^	No inconsistency	Serious indirectness (−1)^o^	No serious imprecision	Unlikely	RR = 1.13 (0.87, 1.47)	1.6% more cutaneous AEs (1.2% fewer to 4.3% more)	⊕⊕⊝⊝, low
Biochemical AE: 9 (1491)	No serious limitation	No inconsistency	Serious indirectness (−1)^o^	Serious imprecision (−1)^l^	Not detected^f^	RR = 1.57 (0.67, 3.66)	2.0% more biochemical AEs (0.2%, 3.8% more)	⊕⊕⊝⊝, low

Abbreviations: AE, adverse effect;GI, gastrointestinal; RR, relative risk.

^a^Relative risk (RR) and rate ratio based on random effects models.

^b^Absolute risk could only be calculated for 4/5 studies related to skin cancers, BCCs and cSCCs (follow-up per individual not available for rate difference calculation in one trial).

^c^No serious risk of bias limitation. Only 2/5 studies reporting BCCs and cSCCs were designed to evaluate skin cancers, and 0/2 trial reporting melanoma was designed to evaluate melanoma (theorical increased risk of selective reporting). However, not downgraded because cancer numbers are not numerical results subject to selection from multiple measurements or analyzes.

^d^No inconsistency. Not downgraded because variability in effect estimates (global *I* ^2^ = 64%) can be explained by risk of bias between trials (subgroup *I* ^2^ = 0% or not applicable).

^e^Serious indirectness. Downgraded from high to moderate because all relevant trials were restricted to tertiary prevention of skin cancers.

^f^Possibility of publication bias not excluded but not considered sufficient to downgrade quality of evidence.

^g^Serious inconsistency. Downgraded from high to moderate because variability in effect estimates (global *I* ^2^ = 53%) not explained in subgroup analyses.

^h^Serious indirectness. Downgraded from moderate to low because all relevant trials were restricted to tertiary prevention.

^i^No inconsistency. Heterogeneity between trials (global *I* ^2^ = 67%) could be explained by variations in daily doses of nicotinamide and risk of bias.

^j^Serious inconsistency. Downgraded from high to moderate because variability in effect estimates (global *I* ^2^ = 61%) not explained in subgroup analyses.

^k^Serious imprecision. Downgraded because null value (MD = 0) is included in 95% CI, and both arms are greater than 25% of relative effect.

^l^Serious imprecision. Downgraded because total number of events < 300, and both arms are greater than 25% of relative effect.

^m^Serious limitations due to 3/21 trials with per-protocol analyzes; 5/21 open label or single blind trials; and inability to judge the risk of selective reporting of adverse effects in 8/21 studies.

^n^Serious limitations due to 4/19 trials with per-protocol analyzes; 6/19 open label or single blind trials; and inability to judge the risk of selective reporting of adverse effects in 6/19 studies.

^o^Serious indirectness. Downgraded from moderate to low because all trials relevant to adverse effects were conducted for other indications than skin cancer chemoprophylaxis.

We observed substantial heterogeneity between trials evaluating skin cancers (global *I* ^2^ = 64%). It could best be explained with subgroups analyses based on risk of bias (*I* ^2^ for subgroup differences = 81.1%). In low risk of bias studies, rate ratio was 0.76 (95% IC, 0.66-0.87; *I* ^2^ = 0%; 414 patients; 2 trials)); in some concerns studies, rate ratio was 0.19 (95% IC, 0.07-0.49; *I* ^2^ = 0%; 106 patients; 2 trials)); in high risk of bias studies, rate ratio was 0.07 (95% IC, 0.00-1.26; *I* ^2^ not applicable; 38 patients; 1 trial) (Figure 3). In subgroup analyses, topical nicotinamide was not found effective in chemoprevention of skin cancers (rate ratio 0.18 (95% IC, 0.02-1.43; *I* ^2^ not applicable; 30 patients; 1 trial)).^38^

## Secondary Outcomes

### Basal Cell Carcinomas

**Figure 4:**
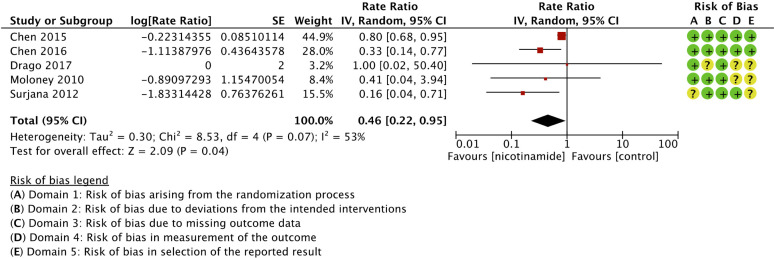
Forest plot and risk of bias for the effect of nicotinamide versus control in basal cell carcinoma chemoprophylaxis.

Nicotinamide was associated with significant reduction in BCCs compared to control (rate ratio 0.46 (95% CI, 0.22-0.95; *I* ^2^ = 53%; 552 patients; 5 trials)).^10,36-39^ Forest plot and risk of bias are presented in Figure 4. Subgroup analyses for BCCs are presented in the Supplemental material. Strength of the evidence for BCCs was judged low due to inconsistency and indirectness (Table 2). Subgroup analyses did not explain heterogeneity between trials (global *I* ^2^ = 53%).

### Cutaneous Squamous Cell Carcinomas

**Figure 5:**
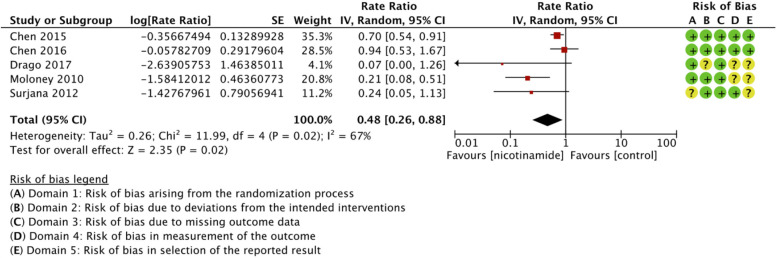
Forest plot and risk of bias for the effect of nicotinamide versus control in cutaneous squamous cell carcinoma chemoprophylaxis.

Nicotinamide was associated with a significant reduction in cSCCs compared to control (rate ratio 0.48 (95% CI, 0.26-0.88; *I* ^2^ = 67%; 552 patients; 5 trials)).^10,36-39^ Forest plot and risk of bias are presented in Figure 5. Subgroup analyses for cSCCs are presented in the Supplemental material. Strength of the evidence was judged moderate due to indirectness (Table 2). Substantial heterogeneity between trials (global *I* ^2^ = 67%) could be explained by variations in daily doses of nicotinamide: the rate ratio for <1 g/day was 0.19 (95% CI, 0.18-0.44; *I* ^2^ = 0%; 88 patients; 2 trials); for ≥1 g/day the rate ratio was 0.48 (95% CI, 0.26-0.88; *I* ^2^ = 30%; 484 patients; 3 trials). Risk of bias could also explain heterogeneity between trials (*I* ^2^ for subgroup differences = 82%; *I* ^2^ for subgroup some concerns = 0%; *I* ^2^ for subgroup low risk = 0%).

### Actinic Keratoses

**Figure 6:**
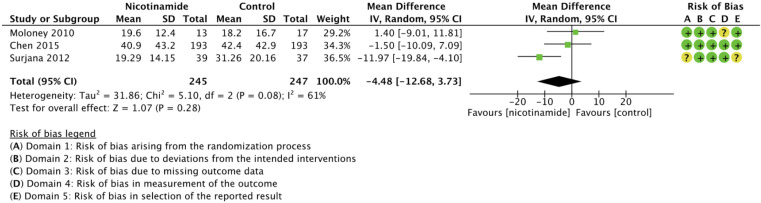
Forest plot and risk of bias for the effect of nicotinamide versus control in actinic keratoses chemoprophylaxis. **g,** gram; **No**, number; NA, not applicable.

No significant difference in means of AK was observed when nicotinamide was compared to control (MD −4.48 (95% CI, −12.68-3.73; *I* ^2^ = 61%; 492 patients; 3 trials)).^10,37,38^ Forest plot and risk of bias are presented in Figure 6. Subgroup analyses for AK are presented in the Supplemental material. Some concerns were brought regarding risk of bias in the randomization process in one trial, where baseline differences in number of AK favor participants randomized to nicotinamide (mean, 36.3; SD 23.8) compared to control (mean, 30.0; SD 19.7).^37^ The strength of the evidence for AK was judged very low because of inconsistency, indirectness, and imprecision (Table 2). Subgroup analyses did not explain heterogeneity between trials. Noteworthy, nicotinamide was associated with a significant reduction in skin cancers in the 3 trials on AK.

### Melanoma

No difference in risk of melanoma was observed with nicotinamide compared to control (RR 0.89 (95% CI, 0.29-2.79; *I* ^2^ = 0%; 416 patients; 2 trials)).^10,38^ Forest plot, risk of bias, and subgroup analyses for melanoma are presented in the Supplemental material. Strength of the evidence was considered low due to indirectness and imprecision (Table 2). Subgroup analyses did not suggest heterogeneity between trials, probably due to the few studies included.

### Digestive Adverse Effects

Nicotinamide was associated with increased risk of digestive AEs compared to control (RR 1.78 (95% CI, 1.30-2.45; *I* ^2^ = 0%; 1859 patients; 21 trials)).^10,26,27,30-37,39,40,42-44,47-51^ Severe diarrhea (undefined) was observed in two trials.^34,51^ Resolution of symptoms was observed after dose diminution from 1 g to 500 mg/day^30^ or therapy withdrawal.^50^ Publication bias is unlikely. Strength of the evidence was very low due to risk of bias, indirectness and imprecision (Table 2). Subgroup analyses revealed no heterogeneity between studies (global *I* ^2^ = 0%). Forest plot, risk of bias, funnel plot, and subgroup analyses for digestive AEs are presented in the Supplemental material.

### Cutaneous Adverse Effects

No differential risks of cutaneous AEs were observed in patients randomized to nicotinamide compared to control (RR 1.13 (95% CI, 0.87-1.47; *I* ^2^ = 0%; 1805 patients; 19 trials)).^10,27,28,31,33,34,36,38,41-48,51-53^ Retained AEs per citation are detailed in eTable 2 in the Supplemental material. Publication bias is unlikely. Forest plot, risk of bias, and funnel plot are presented in the Supplemental material. Strength of the evidence was judged low due to risk of bias and indirectness (Table 2). Subgroup analyses revealed no heterogeneity between studies (global *I* ^2^ = 0) and are presented in the Supplemental material.

### Biochemical Adverse Effects

No differential risks of biochemical AEs were observed with nicotinamide compared to control (RR 1.57 (95% CI, 0.67-3.66; *I* ^2^ = 29%; 1491 patients; 9 trials)).^10,29,30,33,34,36,41,47,50^ We had some concerns regarding the risk of bias in one open-label trial.^33^ Strength of the evidence was judged low due to indirectness and imprecision (Table 2). Heterogeneity between trials was unimportant (global *I* ^2^ = 29%). Residual heterogeneity was partially explained by subgroups analyses. Forest plot, risk of bias, funnel plot, and subgroup analyses for biochemical AEs are presented in the Supplemental material.

## Discussion

Nicotinamide was associated with a significant reduction in new skin cancers (rate ratio 0.50 (95% CI, 0.29-0.85; *I* ^2^ = 64%); moderate-quality evidence), which included data on BCCs, cSCCs, and melanomas. Results from subgroups analyses suggest that nicotinamide could benefit both to organ transplant and immunocompetent patients (*I* ^2^ for subgroup differences = 0%; *I* ^2^ for immunocompetent = 76%; *I* ^2^ for solid organ transplants = 55%). Nicotinamide was also associated with a significant effect on chemoprophylaxis of BCCs (rate ratio 0.46 (95% CI, 0.22-0.95; *I* ^2^ = 53%); low-quality evidence) and cSCCs (rate ratio 0.48 (95% CI, 0.26-0.88; *I* ^2^ = 67%); moderate-quality evidence). The effects of nicotinamide on AK and melanoma were not significant. Risk of digestive AEs significantly increased in patients randomized to nicotinamide compared to control (RR 1.78 (95% CI, 1.30-2.45; *I* ^2^ = 0%); very low-quality evidence).

Recent recommendations published in the Journal of the American Academy of Dermatology support the use of oral nicotinamide 500 mg twice daily in patients with a field of cancerization or >1 previous cSCCs.^8,9^ Our results are consistent with current recommendations on chemoprophylaxis of cSCCs, but they differ regarding other indications of nicotinamide. First, our study allows us to consider chemoprevention of BCCs with nicotinamide, which is not a current indication.^54^ Secondly, our results do not directly support the use of nicotinamide in chemoprophylaxis of AK. The use of oral nicotinamide in chemoprophylaxis of AK could be argued considering significant results in reduction of cSCCs. In a systematic review and meta-analysis of RCTs on solid organ transplant recipients, the effect of nicotinamide in skin cancer chemoprophylaxis was considered uncertain. However, their results should be cautiously interpreted, as they evaluated the effects of multiple interventions without network meta-analysis.^11^ Finally, current American recommendations do not support surveillance of AEs with nicotinamide with the exception of liver failure with doses > 3 g/day.^9^ Our results demonstrate a significant increased risk in digestive AEs in patients randomized to nicotinamide compared to control.

Strengths of our study include large eligibility criteria targeting patients who received nicotinamide regardless of treatment indication and route of administration; implementation of a primary outcome capable to bring all types of skin cancers together; peer-reviewed search strategy without restriction for language or year of publication; appraisal of internal validity using RoB2; strength of the evidence evaluation using GRADE methodology; and subgroup analyses planned *a priori*. Limitations include low number of included trials on the basis of skin cancers, which could have been avoided by a search strategy targeting a population of individuals with a history of skin cancer; evaluation of AEs limited to three categories with quantitative reports, which may overestimate effect measures on AEs; and inclusion of trials conducted with topical nicotinamide, whose pharmacokinetics is still being studied in translational research.^13-15^ Recent advances on cutaneous absorption of nicotinamide supported our decision to use eligibility criteria encompassing topical nicotinamide. Nevertheless, estimates for cutaneous and biochemical AEs were nonsignificant, as was the estimate for topical nicotinamide in skin cancer subgroup analyses.

## Conclusion

Consideration should be given for skin cancer chemoprophylaxis with nicotinamide 500 mg *per os* twice daily for a minimum of 12 months in healthy patients or organ transplant recipients (GRADE: weak recommendation; moderate-quality evidence), in particular for BCCs chemoprophylaxis (GRADE: weak recommendation; low-quality evidence) and cSCCs chemoprophylaxis (GRADE: weak recommendation; moderate-quality evidence). These conclusions should be interpreted keeping in mind that all included trials evaluated the effect of nicotinamide in tertiary prevention of skin cancers. Effect of nicotinamide require further evaluation in regard to chemoprevention of AK and melanoma, potential long-term benefits, safety in patients with comorbidities such as chronic kidney disease, and enduring effects after its discontinuation. Low cost and over-the-counter accessibility of nicotinamide support its relevance in tertiary prevention of skin cancers.

## Supplemental Material

Supplementary Material 1 - Supplemental material for Effect of Nicotinamide in Skin Cancer and Actinic Keratoses Chemoprophylaxis, and Adverse Effects Related to Nicotinamide: A Systematic Review and Meta-Analysis Click here for additional data file. Supplemental material, Supplementary Material 1, for Effect of Nicotinamide in Skin Cancer and Actinic Keratoses Chemoprophylaxis, and Adverse Effects Related to Nicotinamide: A Systematic Review and Meta-Analysis by Laurence Mainville, Anne-Sophie Smilga and Paul R. Fortin in Journal of Cutaneous Medicine & Surgery
