# Supplementary material for: Effect of Nicotinamide in Skin Cancer and Actinic Keratoses Chemoprophylaxis, and Adverse Effects Related to Nicotinamide: A Systematic Review and Meta-Analysis
Source: J Cutan Med Surg. 2022 Feb 8;26(3):297–308. doi: 10.1177/12034754221078201 (PMC9125143; doi:10.1177/12034754221078201)
Supplement: Supplementary Material 1 - Supplemental material for Effect of Nicotinamide in Skin Cancer and Actinic Keratoses Chemoprophylaxis, and Adverse Effects Related to Nicotinamide: A Systematic Review and Meta-Analysis [file sj-pdf-1-cms-10.1177_12034754221078201.pdf]

Supplemental material

eTable 1. Medline Search strategy

eTable 2. Digestive, cutaneous, and biochemical adverse events retained for analyses

eFigure 1. Subgroup analyses for secondary outcome: Number of new basal cell carcinomas

eFigure 2. Subgroup analyses for secondary outcome: Number of new cutaneous squamous cell carcinomas

eFigure 3. Subgroup analyses for secondary outcomes: Number of actinic keratoses

eFigure 4. Forest plot, risk of bias, and subgroup analyses for secondary outcomes: Melanoma

eFigure 5. Forest plot, risk of bias, funnel plot, and subgroup analyses for secondary outcome: Digestive adverse effects

eFigure 6. Forest plot, risk of bias, funnel plot, and subgroup analyses for secondary outcome: Cutaneous adverse effects

eFigure 7. Forest plot, risk of bias, funnel plot and subgroup analyses for secondary outcome: Biochemical adverse effects

eTable1. Medline Search strategy

**Date of last search : 06-10-2020**<br>**Limit:** none<br>**ECR Filter:** *Cochrane Handbook for Systematic Reviews of Interventions,* Chapter 4			
**Concepts**	**#**	**Search stategy**	**Results**
Nicotinamide	1	“Niacinamide”[Mesh:noexp]	12 502
	2	“Nicotinamide”[Title/Abstract]	21 898
	3	“Niacinamide”[Title/Abstract]	531
	4	“Vitamin B3”[Title/Abstract]	382
	5	“B3 Vitamin”[Title/Abstract]	11
	6	“Vitamin B 3”[Title/Abstract]	99
	7	“b 3 vitamin”[Title/Abstract]	1
	8	“3 pyridinecarboxamide”[Title/Abstract]	45
	9	“Vitamin PP”[Title/Abstract]	148
	10	“Enduramide”[Title/Abstract]	1
	11	“Nicobion”[Title/Abstract]	1
	12	“Nicotinic amide”[Title/Abstract]	23
	13	“Nicotinic acid amide” [Title/Abstract]	59
	14	“3-carbamoylpyridine”[Title/Abstract]	1
Nicotinamide (combined)	15	#1 OR #2 OR #3 OR #4 OR #5 OR #6 OR #7 OR #8 OR #9 OR #10 OR #11 OR #12 OR #13 OR #14	**30 717**
Randomized controlled trials	16	Randomized controlled trial[Publication Type]	516 470
	17	Controlled clinical trial[Publication Type]	605 500
	18	Randomized[Title/Abstract]	537 939
	19	Placebo[Title/Abstract]	217 737
	20	Clinical trials as topic[MeSH Terms]	347 508
	21	Randomly[Title/Abstract]	343 681
	22	Trial[Title]	226 935
	23	Animals[MeSH Terms] NOT Humans[MeSH Terms])	4 746 125
Randomized controlled trials (combined)	24	#16 OR #17 OR #18 #19 OR #20 OR #21 OR #22 NOT #23	**1 300 927**
Combination of concepts	25	#15 AND #24	**1 620**

eTable 2. Digestive, cutaneous, and biochemical adverse events retained for analyses

Trial	**Digestive adverse event**	**Cutaneous adverse event**	**Biochemical adverse event**
Chouinard 1979	Constipation	None	None
Hulshof 1987	Pain in stomach	Skin rash	None
Fivenson 1994	GI upset	Multiple decubitus ulcers	None
Shalita 1995	None	Local reaction at application site	None
Jonas 1996	GI disturbances (eructation, nausea, or loose stools)	None	None
Gale 2004	None	None	Increased alanine transaminase, aspartate transaminase, or gamma glutamyl transpeptidase (>3x the upper limit)
Sun 2007	Constipation	None	None
Young 2009	Severe diarrhea	Pruritic rash, flushing	None
Jerajani 2010	None	Transient burning sensation following application	None
Moloney 2010	None	Intermittent itch	None
Shahbazian 2011	Diarrhea, resolved by lowering NAM	-	Thrombocytopenia
Allam 2012	GI disturbance and diarrhea	Flushing and abdominal rash	None
Surjana 2012	Nausea	None	None
Khodaeiani 2013	None	Mild burning	None
Pop-Busui 2013	None	Skin rash	Elevated liver enzymes
Fabbrocini 2014	None	Burning sensation and pruritus	None
Chen 2015	Diarrhea	Skin and subcutaneous tissue disorders	Investigations – Other
Watanabe 2015	None	Scaling	None
Chen 2016	Diarrhea	Skin ulceration	Creatinine increased
El Borolossy 2016	Diarrhea	Flushing	Decrease in platelet count (not analysed due to lack of data to dichotomize)
Kasliwal 2016	GI discomfort	None	None
Drago 2017	Diarrhea	None	None
Lenglet 2017	Digestion impaired	Prutitis rash	Thrombocytopenia, resolved rapidly after withdrawal of NAM (<4 weeks)
Rucklidge 2018	GI disturbances	Rash	None

Trial	**Digestive adverse event**	**Cutaneous adverse event**	**Biochemical adverse event**
Caetano 2019	GI disturbances	Skin and subcutaneous tissue disorders	None
Ix 2019	Severe diarrhea	Bruising	Thrombocytopenia <100,000/mm^2
El Ters 2020	Gastroesophagial reflux	Flushing	Anemia
Hui 2020	GI discomfort	None	None
Liu 2020	Nausea/vomiting/diarrhea resolving rapidly after withdrawal of NAM (<3 weeks)	None	Thrombocytopenia, rapidly resolved after withdrawal of NAM (<3 weeks); 75% (3/4) cases were detected <8 weeks from start of NAM; lowest platelet count was 75,000/mm^2

GI, Gastrointestinal; **NAM,** Nicotinamide.

eFigure 1. Forest plot, risk of bias, and subgroup analyses for secondary outcome: Number of new basal cell carcinomas

Patient group*	Nicotinamide		Control		I^2	Rate ratio (95% CI)
	No of patients	No of new BCCs	No of patients	No of new BCCs		
Overall	275	249	277	363	53%	
Route of administration					0%†	
Enteral	262	248	260	360	64%	
Topical	13	1	17	3	NA	
Daily dose					0%†	
<1g	32	1	36	3	0%	
1g and more	243	248	241	360	76%	
Treatment duration					86.4%†	
<1 year	82	10	84	36	0%	
1 year and more	193	239	193	327	NA	
Risk of skin cancer					0%†	
General	245	242	247	342	57%	
Predisposing condition	30	7	30	21	0%	
Risk of bias					0%†	
Low risk	204	246	204	348	75%	
Some concerns	52	3	54	15	0%	
High risk	19	0	19	0	NA	

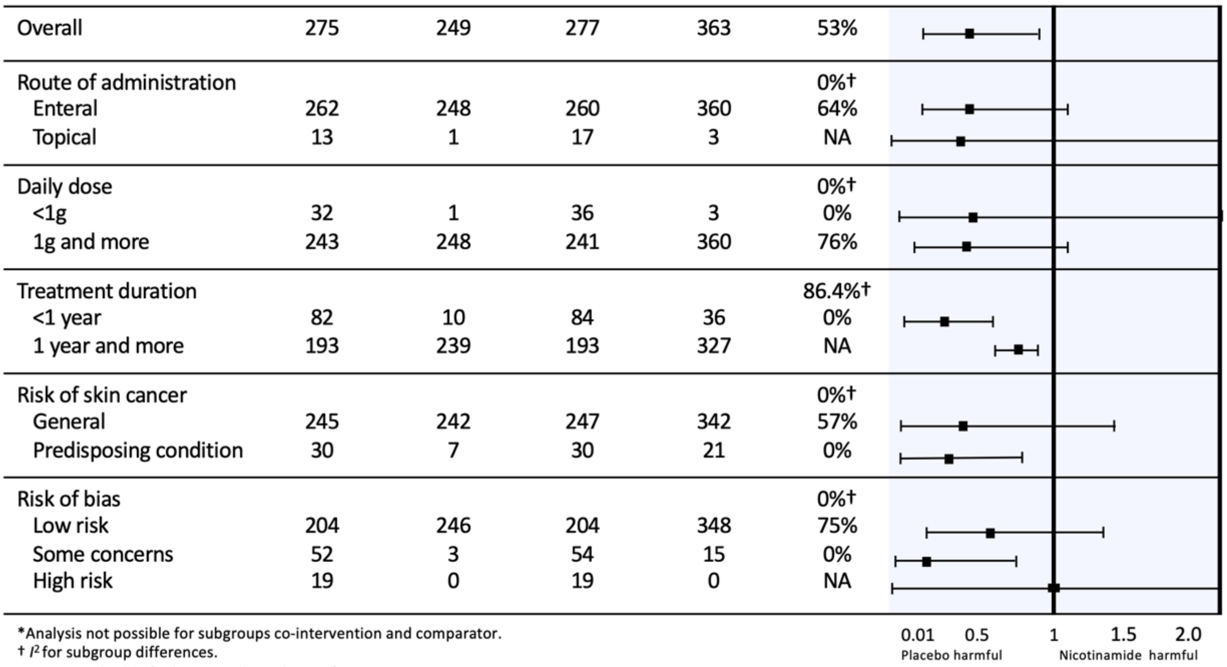

*Analysis not possible for subgroups co-intervention and comparator.
† I^2 for subgroup differences.
NA, not applicable (only one trial in subgroup).

eFigure 2. Forest plot, risk of bias, and subgroup analyses for secondary outcome: Number of new cutaneous squamous cell carcinomas

Patient group*	Nicotinamide		Control		I^2	Rate ratio (95% CI)
	No of patients	No of new SCCs	No of patients	No of new SCCs		
Overall	275	122	277	178	67%	
Route of administration					79.7%†	
Enteral	262	248	260	360	43%	
Topical	13	1	17	3	NA	
Daily dose					87.4%†	
<1g	32	1	36	3	0%	
1g and more	243	248	241	360	30%	
Treatment duration					41.5%†	
<1 year	82	10	84	36	73%	
1 year and more	193	239	193	327	NA	
Risk of skin cancer					0%†	
General	245	242	247	342	75%	
Predisposing condition	30	7	30	21	67%	
Risk of bias					82%†	
Low risk	204	246	204	348	0%	
Some concerns	52	3	54	15	0%	
High risk	19	0	19	0	NA	

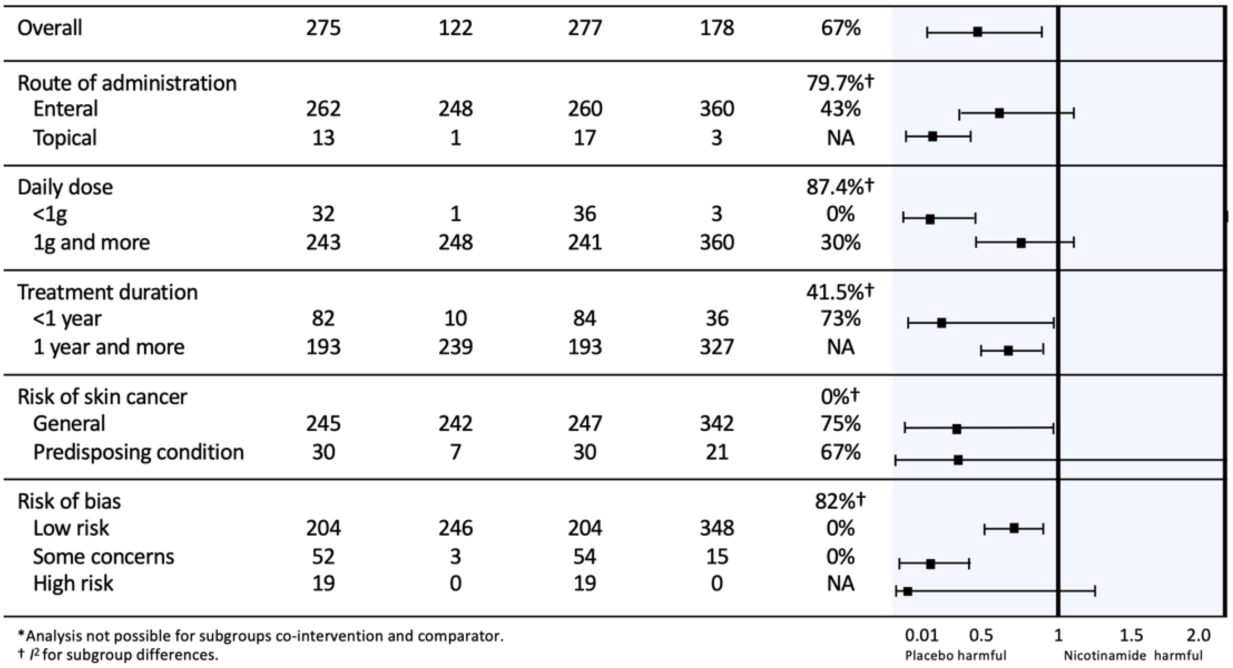

*Analysis not possible for subgroups co-intervention and comparator.
† I^2 for subgroup differences.
NA, not applicable (only one trial in subgroup).

eFigure 3. Forest plot, risk of bias, and subgroup analyses for secondary outcomes: Number of actinic keratoses

Patient group*	Nicotinamide		Control		I^2
	No of patients	Mean (STD) of AK	No of patients	Mean (STD) of AK	
Overall	245	36.3 (38.8)	247	39.1 (38.9)	61%
Route of administration					12.3%†
Enteral	232	37.3 (39.8)	230	40.4 (40.1)	69%
Topical	13	19.6 (12.4)	17	18.2 (17.7)	NA
Daily dose					18.9%†
<1g	13	19.6 (12.4)	17	18.2 (17.7)	NA
1g and more	232	37.3 (39.8)	230	40.4 (40.1)	68%
Treatment duration					0%†
<1 year	52	19.5 (12.7)	54	27.1 (19.0)	75%
1 year and more	193	40.9 (43.2)	193	42.4 (42.9)	NA
Risk of bias					79.7%†
Low risk	206	39.6 (41.9)	210	40.4 (41.4)	0%
Some concerns	39	19.3 (14.2)	37	31.3 (20.2)	NA
High risk	0	-	0	-	-

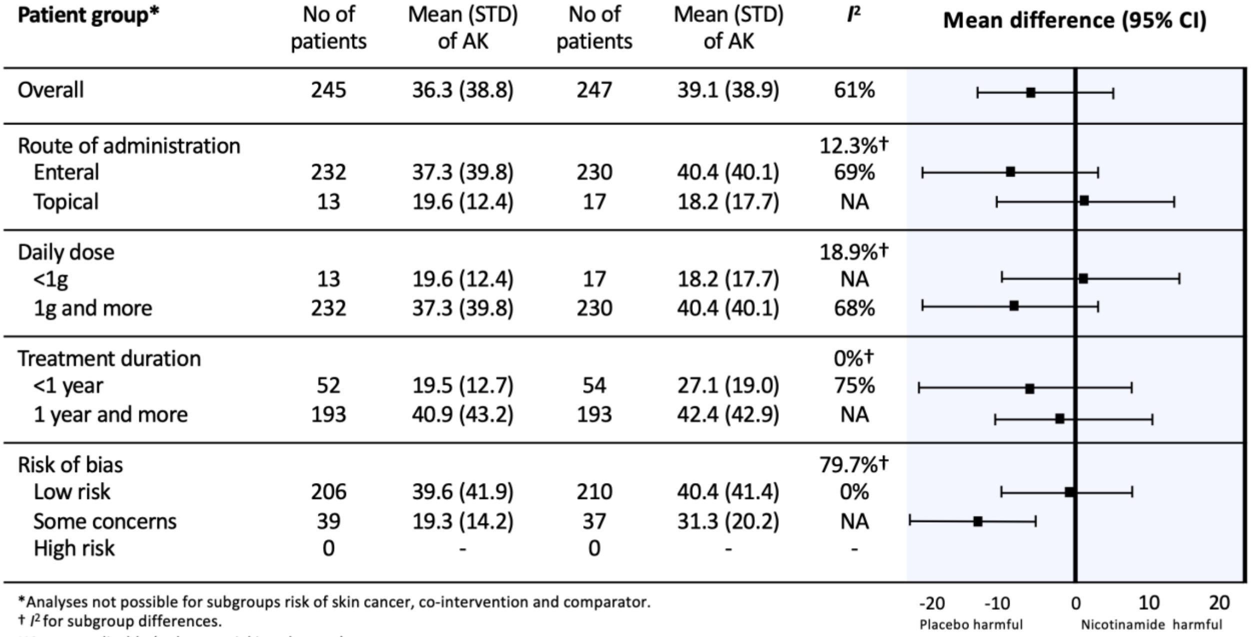

*Analyses not possible for subgroups risk of skin cancer, co-intervention and comparator.
† I^2 for subgroup differences.
NA, not applicable (only one trial in subgroup).

eFigure 4. Forest plot, risk of bias, and subgroup analyses for secondary outcomes: Melanoma

Study or Subgroup	Nicotinamide Events	Nicotinamide Total	Control Events	Control Total	Weight	Risk Ratio IV, Random, 95% CI	Risk of Bias A B C D E
Chen 2015	5	193	5	193	86.7%	1.00 [0.29, 3.40]	+ + + + +
Moloney 2010	0	13	1	17	13.3%	0.43 [0.02, 9.74]	+ + + ? ?
**Total (95% CI)**		**206**		**210**	**100.0%**	**0.89 [0.29, 2.79]**	
Total events	5		6				

Heterogeneity: Tau² = 0.00; Chi² = 0.25, df = 1 (P = 0.62); I² = 0%

Test for overall effect: Z = 0.19 (P = 0.85)

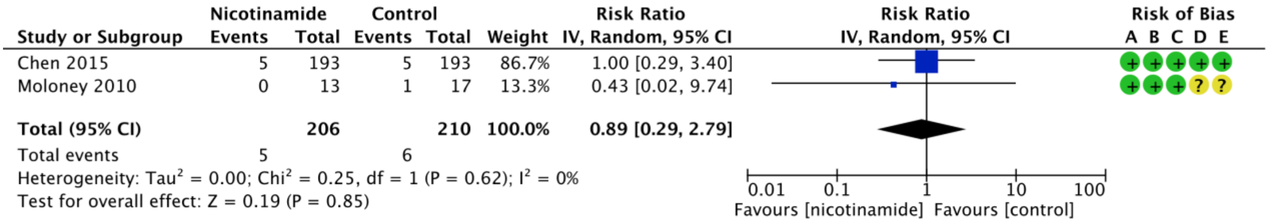

Risk of bias legend
(**A**) Domain 1: Risk of bias arising from the randomization process
(**B**) Domain 2: Risk of bias due to deviations from the intended interventions
(**C**) Domain 3: Risk of bias due to missing outcome data
(**D**) Domain 4: Risk of bias in measurement of the outcome
(**E**) Domain 5: Risk of bias in selection of the reported result

Patient group*	Nicotinamide No of patients	Nicotinamide Melanoma	Control No of patients	Control Melanoma	I^2
Overall	206	5	210	6	0%
Route of administration					0%†
Enteral	193	5	193	5	NA
Topical	13	0	17	1	NA
Daily dose					0%†
<1g	13	0	17	1	NA
1g and more	193	5	193	5	NA
Treatment duration					0%†
<1 year	13	0	17	1	NA
1 year and more	193	5	193	5	NA
Risk of bias					0%†
Low risk	193	5	193	5	NA
Some concerns	13	0	17	0	NA
High risk	0	0	0	0	-

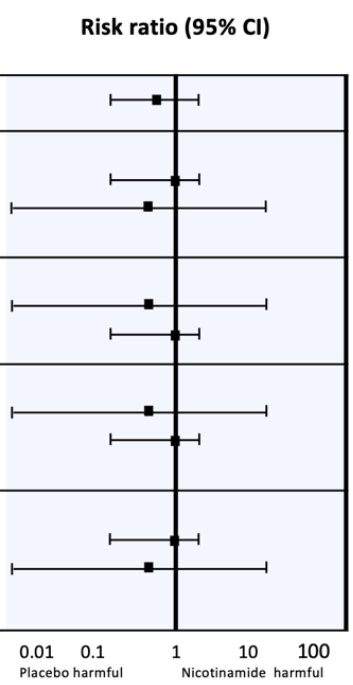

*Analysis not possible for subgroups risk of skin cancer, co-intervention and comparator.
† I^2 for subgroup differences.
NA, not applicable (only one trial in subgroup).

eFigure 5. Forest plot, risk of bias, funnel plot, and subgroup analyses for secondary outcome: Digestive adverse effects

Study or Subgroup	Nicotinamide Events	Nicotinamide Total	Control Events	Control Total	Weight	Risk Ratio IV, Random, 95% CI	Risk of Bias A	B	C	D	E
Allam-2012	3	30	0	30	1.2%	7.00 [0.38, 129.93]	+	?	?	?	?
Caetano 2019	1	88	0	44	1.0%	1.52 [0.06, 36.49]	+	+	+	+	?
Chen 2015	9	193	4	193	7.5%	2.25 [0.70, 7.18]	+	+	+	+	+
Chen 2016	3	11	1	11	2.3%	3.00 [0.37, 24.58]	+	+	+	+	+
Chouinard 1979	7	17	2	8	5.7%	1.65 [0.44, 6.21]	+	+	+	+	?
Drago 2017	1	19	0	19	1.0%	3.00 [0.13, 69.31]	+	?	+	?	?
El Borolossy 2016	10	30	0	30	1.3%	21.00 [1.29, 342.93]	+	+	+	?	+
El Ters 2020	7	18	3	18	7.2%	2.33 [0.71, 7.63]	+	+	+	+	+
Fivenson 1994	2	12	0	6	1.2%	2.69 [0.15, 48.64]	+	?	+	?	?
Hui 2020	3	30	2	27	3.5%	1.35 [0.24, 7.48]	+	?	+	+	+
Huishof 1987	1	24	1	24	1.4%	1.00 [0.07, 15.08]	+	+	+	+	?
Ix-2019	20	104	12	101	23.1%	1.62 [0.84, 3.14]	+	+	+	+	+
Jonas 1996	12	31	8	29	18.6%	1.40 [0.67, 2.93]	+	+	+	+	?
Kasliwal 2016	2	96	2	95	2.7%	0.99 [0.14, 6.88]	+	?	+	+	+
Lenglet 2017	1	49	0	51	1.0%	3.12 [0.13, 74.80]	+	?	+	?	+
Liu 2020	4	49	2	49	3.7%	2.00 [0.38, 10.42]	+	+	+	+	+
Rucklidge 2018	9	47	7	46	12.5%	1.26 [0.51, 3.10]	+	+	+	+	+
Shabahzian 2011	4	24	0	24	1.2%	9.00 [0.51, 158.52]	+	+	+	+	+
Sun 2007	2	45	1	44	1.8%	1.96 [0.18, 20.80]	+	+	+	+	+
Surjana 2012	1	39	0	37	1.0%	2.85 [0.12, 67.83]	?	+	+	+	?
Young 2009	1	8	0	9	1.1%	3.33 [0.15, 71.90]	+	+	+	+	+
**Total (95% CI)**		**964**		**895**	**100.0%**	**1.78 [1.30, 2.45]**					
Total events	103		45								

Heterogeneity: $Tau^2 = 0.00$; $Chi^2 = 7.94$, df = 20 (P = 0.99); $I^2 = 0\%$

Test for overall effect: Z = 3.57 (P = 0.0004)

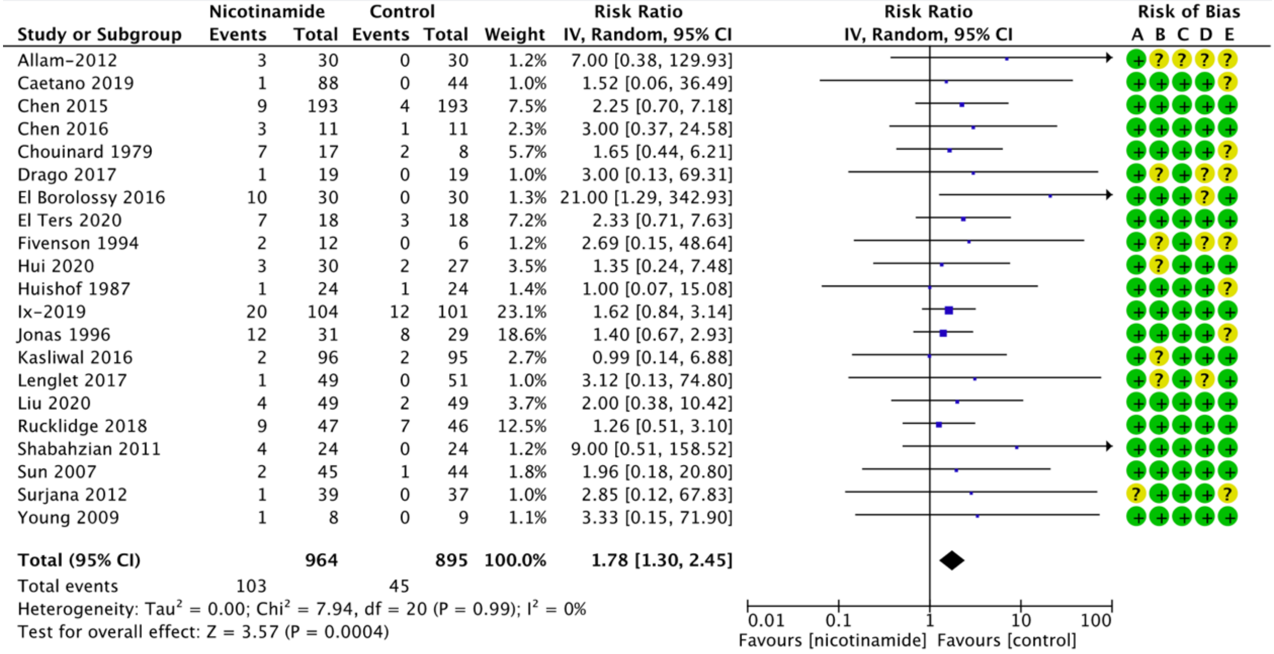

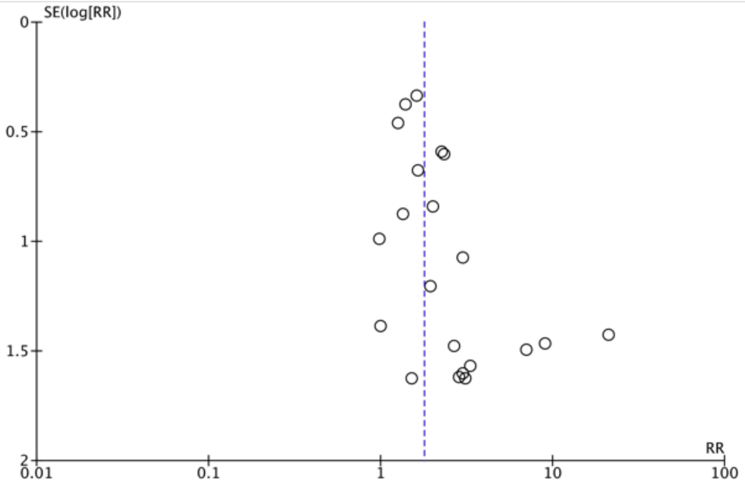

eFigure 5. Forest plot, risk of bias, funnel plot, and subgroup analyses for secondary outcome: Digestive adverse effects *(continued)*

Patient group*	Nicotinamide		Placebo		I^2
	No of patients	Digestive AE	No of patients	Digestive AE	
Overall	964	103	895	45	0%
Route of administration					0%†
Enteral	876	102	851	45	0%
Topical	88	1	44	0	NA
Daily dose					0%†
<1g	403	33	356	11	0%
1g and more	561	70	539	34	0%
Treatment duration					0%†
<1 year	588	61	528	24	0%
1 year and more	376	42	367	21	0%
Risk of skin cancer					0%†
General	934	99	865	44	0%
Predisposing condition	30	4	30	1	0%
Co-intervention					0%†
Absence	495	47	491	21	0%
Presence	469	56	404	24	0%
Risk of bias					0%†
Low risk	499	59	495	30	0%
Some concerns	435	41	370	15	0%
High risk	30	3	30	0	NA

Risk ratio (95% CI)

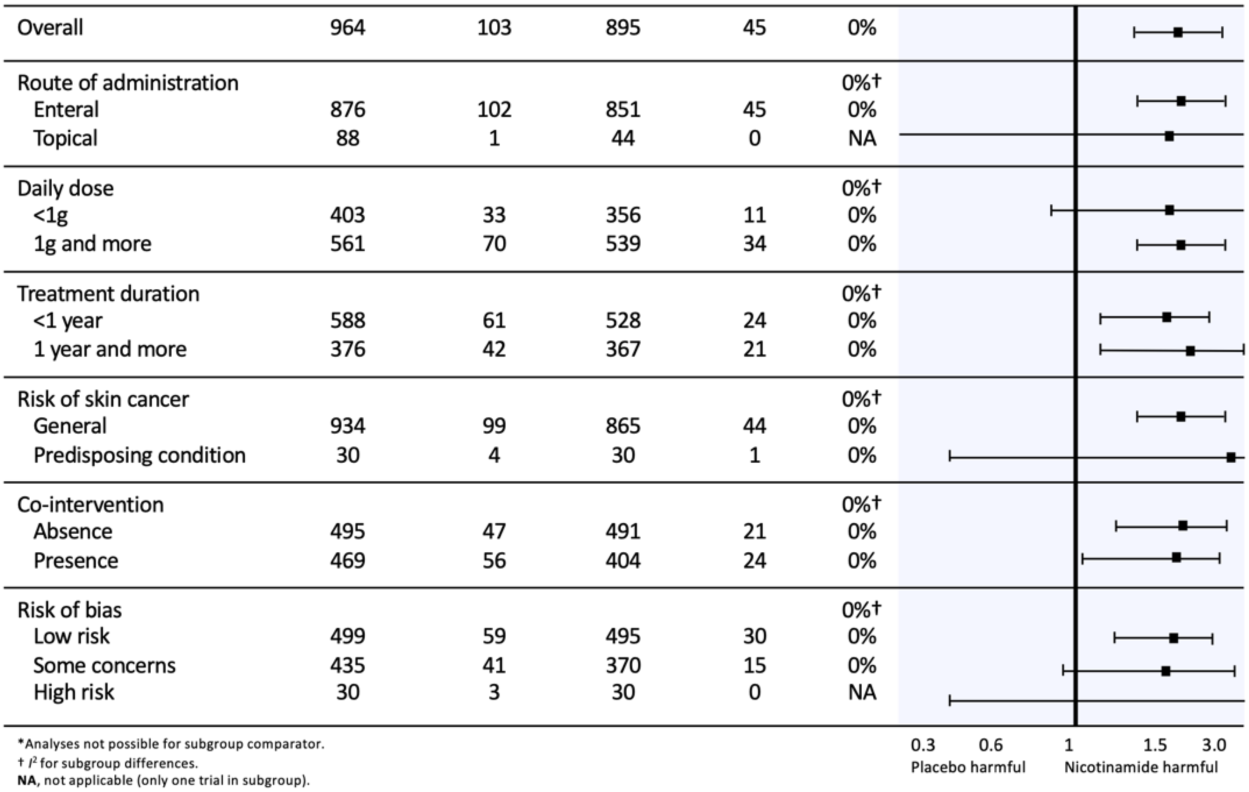

*Analyses not possible for subgroup comparator.
† I^2 for subgroup differences.
NA, not applicable (only one trial in subgroup).

eFigure 6. Forest plot, risk of bias, funnel plot, and subgroup analyses for secondary outcome: Cutaneous adverse effects

Study or Subgroup	Nicotinamide Events	Nicotinamide Total	Control Events	Control Total	Weight	Risk Ratio IV, Random, 95% CI	Risk of Bias A	B	C	D	E
Allam-2012	2	30	0	30	0.8%	5.00 [0.25, 99.95]	+	?	?	?	?
Caetano 2019	0	88	1	44	0.7%	0.17 [0.01, 4.05]	+	+	+	+	?
Chen 2015	9	193	11	193	9.2%	0.82 [0.35, 1.93]	+	+	+	+	+
Chen 2016	1	11	1	11	1.0%	1.00 [0.07, 14.05]	+	+	+	+	+
El Borolossy 2016	8	30	0	30	0.9%	17.00 [1.03, 281.91]	+	+	+	?	+
El Ters 2020	2	18	2	18	2.0%	1.00 [0.16, 6.35]	+	+	+	+	+
Fabbrocini 2014	2	24	0	24	0.8%	5.00 [0.25, 98.96]	+	?	+	?	?
Fivenson 1994	0	12	1	6	0.7%	0.18 [0.01, 3.85]	+	?	+	?	?
Huishof 1987	0	24	1	24	0.7%	0.33 [0.01, 7.80]	+	+	+	+	?
Ix-2019	41	104	34	101	51.4%	1.17 [0.81, 1.68]	+	+	+	+	+
Jerajani 2010	6	124	2	122	2.7%	2.95 [0.61, 14.34]	+	+	+	+	?
Khodaeiani 2013	7	40	2	40	3.0%	3.50 [0.77, 15.83]	+	+	+	+	+
Lenglet 2017	1	49	0	51	0.7%	3.12 [0.13, 74.80]	+	?	+	?	+
Moloney 2010	0	13	1	17	0.7%	0.43 [0.02, 9.74]	+	+	+	?	+
Pop-Busui 2013	6	22	6	22	7.3%	1.00 [0.38, 2.62]	+	+	+	+	+
Rucklidge 2018	4	47	6	46	4.7%	0.65 [0.20, 2.16]	+	+	+	+	+
Shalita 1995	10	38	9	38	11.1%	1.11 [0.51, 2.42]	+	?	+	+	+
Watanabe 2015	0	52	3	52	0.8%	0.14 [0.01, 2.70]	+	?	+	+	+
Young 2009	1	8	1	9	1.0%	1.13 [0.08, 15.19]	+	+	+	+	+
**Total (95% CI)**		**927**		**878**	**100.0%**	**1.13 [0.87, 1.47]**					
Total events	100		81								

Heterogeneity: $Tau^2 = 0.00$; $Chi^2 = 16.53$, df = 18 (P = 0.56); $I^2 = 0\%$

Test for overall effect: Z = 0.95 (P = 0.34)

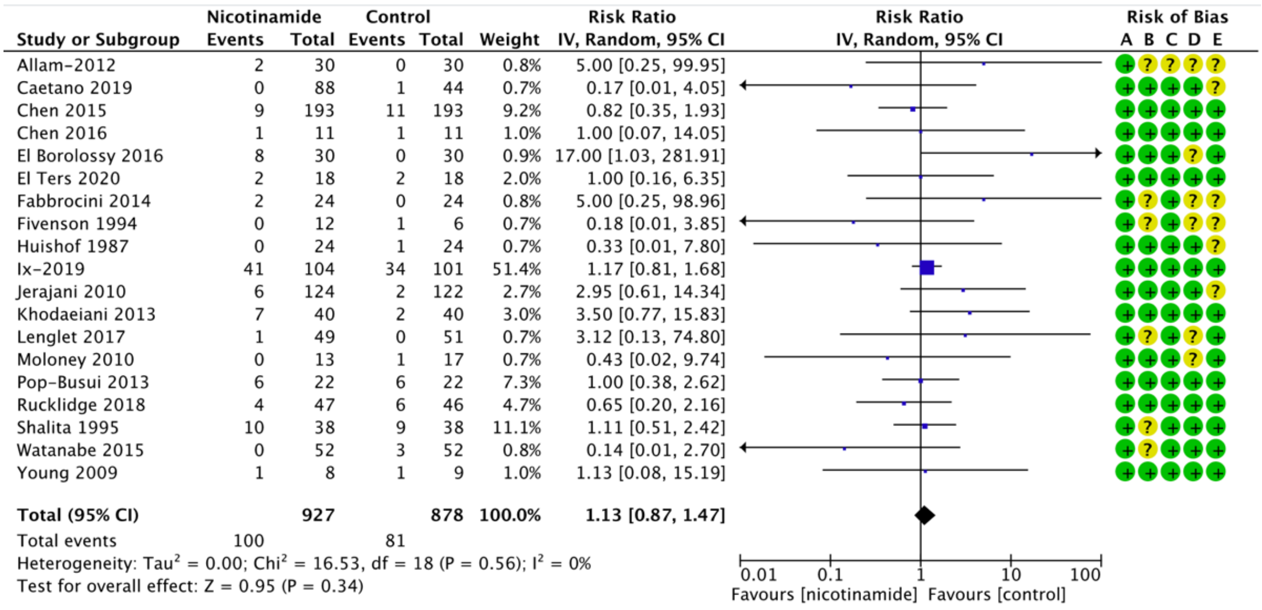

Risk of bias legend

(**A**) Domain 1: Risk of bias arising from the randomization process

(**B**) Domain 2: Risk of bias due to deviations from the intended interventions

(**C**) Domain 3: Risk of bias due to missing outcome data

(**D**) Domain 4: Risk of bias in measurement of the outcome

(**E**) Domain 5: Risk of bias in selection of the reported result

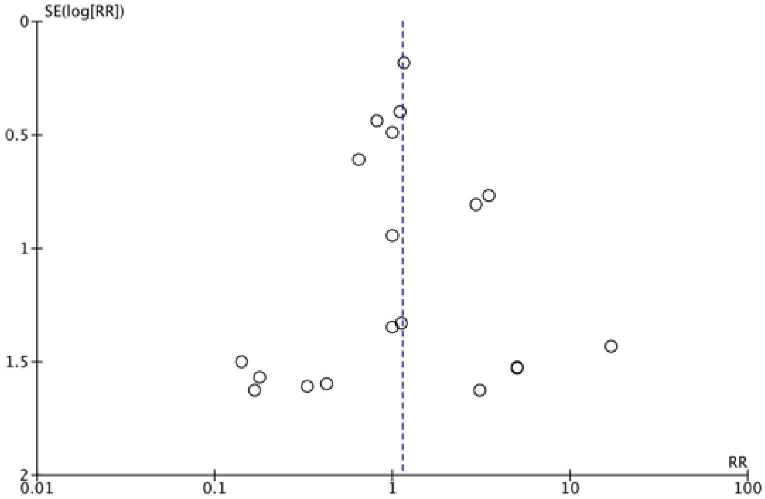

eFigure 6. Forest plot, risk of bias, funnel plot, and subgroup analyses for secondary outcome: Cutaneous adverse effects (*continued)*

Patient group*	Nicotinamide		Placebo		I^2	Risk ratio (95% CI)
	No of patients	Cutaneous AE	No of patients	Cutaneous AE		
Overall	927	100	878	81	0%	
Route of administration					0%†	
Enteral	548	75	541	63	0%	
Topical	379	25	337	18	24%	
Daily dose					0%†	
<1g	510	39	467	25	28%	
1g and more	417	61	411	56	0%	
Treatment duration					0%†	
<1 year	578	42	538	27	8%	
1 year and more	349	58	340	54	0%	
Risk of skin cancer					0%†	
General	916	99	867	80	0%	
Predisposing condition	11	1	11	1	NA	
Co-intervention					0%†	
Absence	594	39	599	33	0%	
Presence	333	61	279	48	27%	
Risk of bias					0%†	
Low risk	443	71	440	63	0%	
Some concerns	454	27	408	18	25%	
High risk	30	2	30	0	NA	

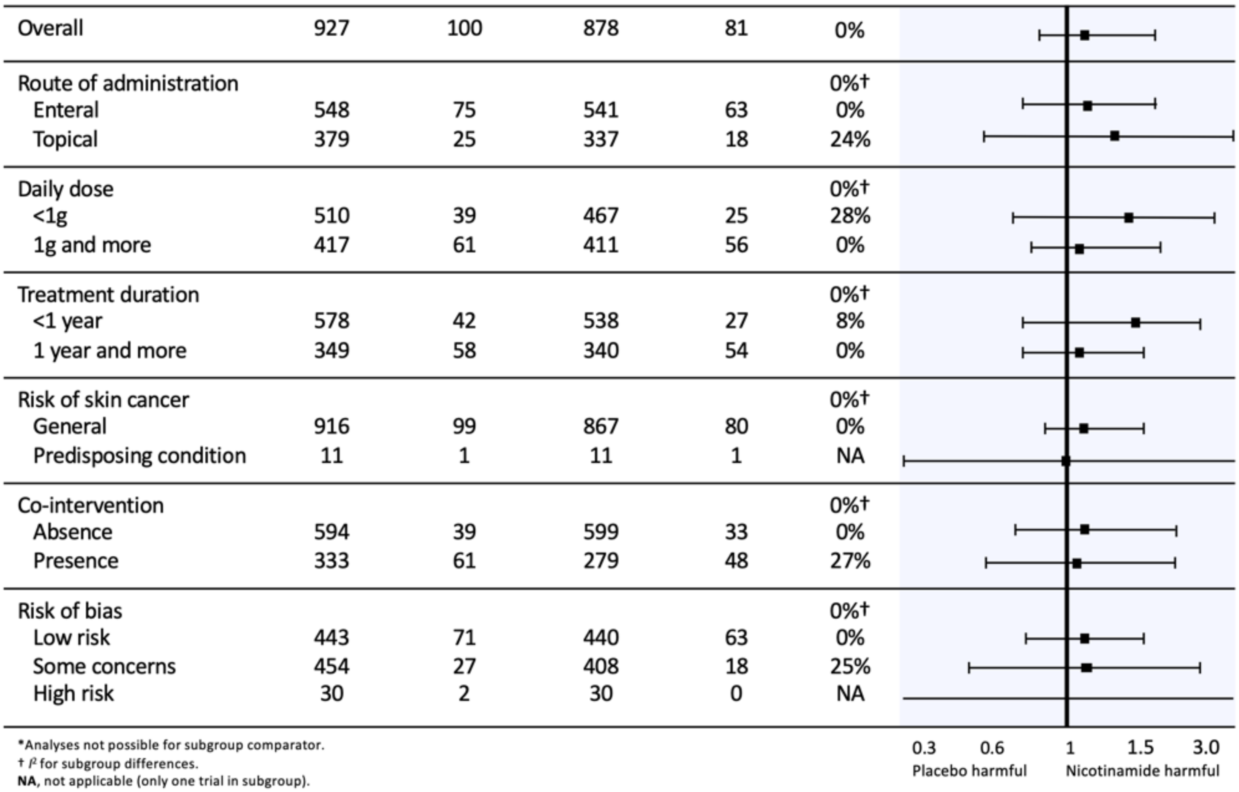

*Analyses not possible for subgroup comparator.
† I^2 for subgroup differences.
NA, not applicable (only one trial in subgroup).

eFigure 7. Forest plot, risk of bias, funnel plot and subgroup analyses for secondary outcome: Biochemical adverse effects

	Nicotinamide		Control			Risk Ratio	Risk of Bias				
Study or Subgroup	Events	Total	Events	Total	Weight	IV, Random, 95% CI	A	B	C	D	E
Chen 2015	4	193	7	193	22.4%	0.57 [0.17, 1.92]	+	+	+	+	+
Chen 2016	3	11	0	11	7.3%	7.00 [0.40, 121.39]	+	+	+	+	+
El Ters 2020	1	18	2	18	10.2%	0.50 [0.05, 5.04]	+	+	+	+	+
Gale 2004	5	276	4	276	20.9%	1.25 [0.34, 4.61]	+	+	+	+	+
Ix-2019	1	104	2	101	9.7%	0.49 [0.04, 5.27]	+	+	+	+	+
Lenglet 2017	4	49	0	51	7.1%	9.36 [0.52, 169.40]	+	?	+	+	+
Liu 2020	4	49	0	49	7.1%	9.00 [0.50, 162.80]	+	+	+	+	+
Pop-Busui 2013	1	22	1	22	7.9%	1.00 [0.07, 15.00]	+	+	+	+	+
Shabahzian 2011	8	24	0	24	7.5%	17.00 [1.04, 278.94]	+	+	+	+	+
**Total (95% CI)**		**746**		**745**	**100.0%**	**1.57 [0.67, 3.66]**					
Total events	31		16								

Heterogeneity: $Tau^2 = 0.45$; $Chi^2 = 11.20$, df = 8 (P = 0.19); $I^2 = 29\%$

Test for overall effect: Z = 1.04 (P = 0.30)

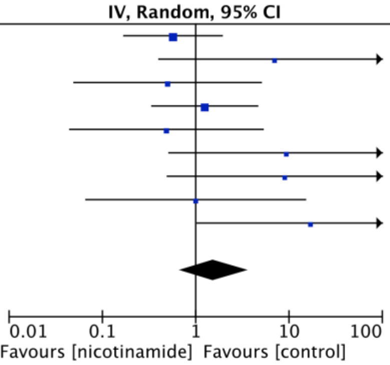

Risk of bias legend

(**A**) Domain 1: Risk of bias arising from the randomization process

(**B**) Domain 2: Risk of bias due to deviations from the intended interventions

(**C**) Domain 3: Risk of bias due to missing outcome data

(**D**) Domain 4: Risk of bias in measurement of the outcome

(**E**) Domain 5: Risk of bias in selection of the reported result

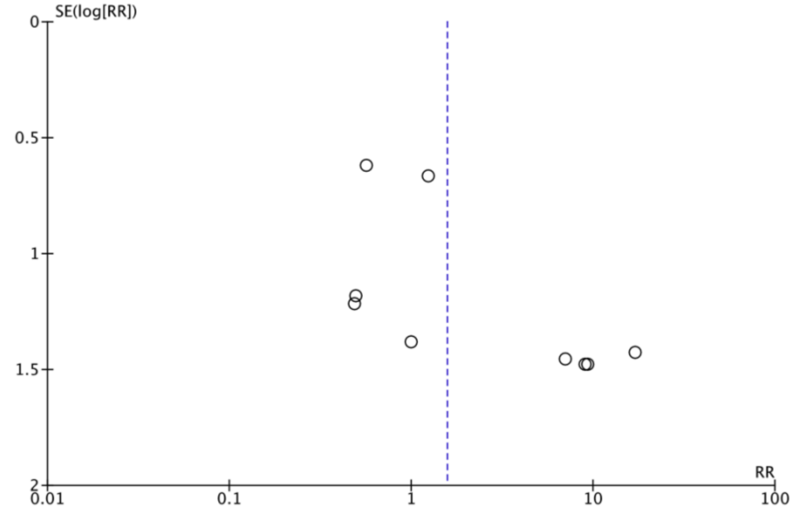

eFigure 7. Forest plot, risk of bias, funnel plot and subgroup analyses for secondary outcome: Biochemical adverse effects *(continued)*

Patient group*	Nicotinamide		Placebo		I^2	Risk ratio (95% CI)
	No of patients	Biochemical AE	No of patients	Biochemical AE		
Overall	746	31	745	16	29%	
Treatment duration					86.3%†	
<1 year	84	15	86	0	0%	
1 year and more	662	16	659	16	0%	
Risk of skin cancer					11.5%†	
General	735	28	734	16	29%	
Predisposing condition	11	3	11	0	NA	
Co-intervention					12.8%†	
Absence	620	29	622	13	42%	
Presence	126	2	123	3	0%	
Risk of bias					37.4%†	
Low risk	697	27	694	16	25%	
Some concerns	49	4	51	0	NA	
High risk	0	0	0	0	-	

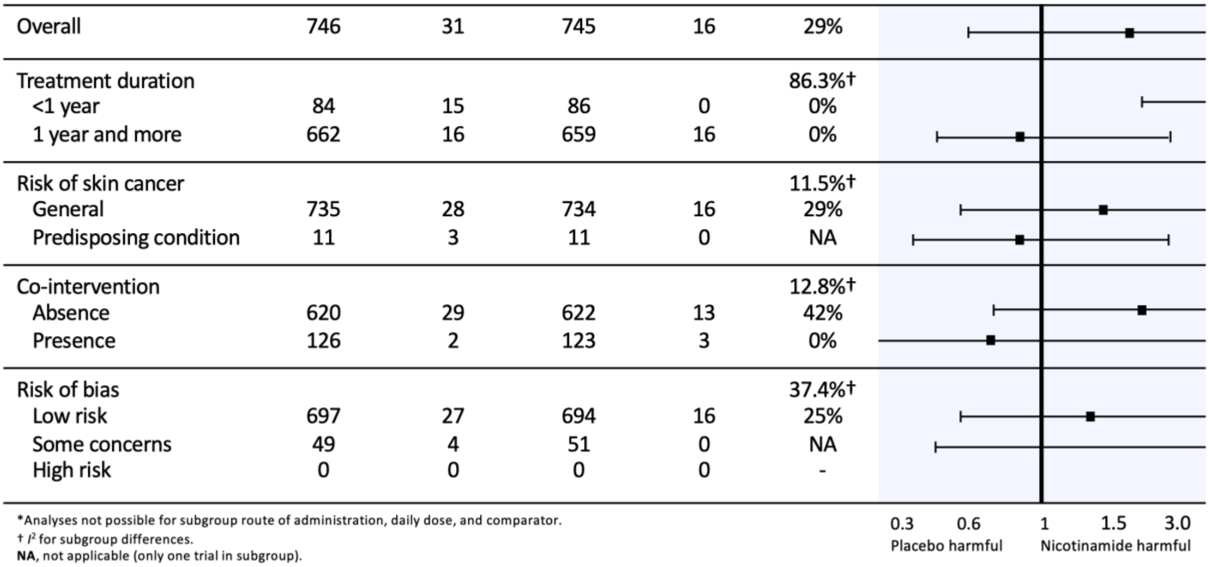

*Analyses not possible for subgroup route of administration, daily dose, and comparator.
† I^2 for subgroup differences.
NA, not applicable (only one trial in subgroup).
